# Supplementary material for: Electrospinning Silk Fibroin/Graphene Nanofiber Membrane Used for 3D Wearable Pressure Sensor
Source: Polymers (Basel). 2022 Sep 16;14(18):3875. doi: 10.3390/polym14183875 (PMC9502556; doi:10.3390/polym14183875)
Supplement: Supplementary file 1 [file polymers-14-03875-s001.zip › polymers-1894783-supplementary.pdf]

Appendix A. Supplementary data

Electrospinning Silk fibroin/Graphene Nanofiber Membrane used for 3D Wearable Pressure Sensor

Zulan Liu [1, 2, †], Jiaxuan Wang [2, †], Qian Zhang [1], Zheng Li [1], Zhi Li [1, 2], Lan Cheng [1, 2, *], Fangyin Dai [1, 2,*]

[1] State Key Laboratory of Silkworm Genome Biology, Key Laboratory of Sericultural Biology and Genetic Breeding, Ministry of Agriculture, Southwest University, Chongqing 400715, China.

[2] Chongqing Engineering Research Center of Biomaterial Fiber and Modern Textile, College of sericulture, Textile and Biomass sciences, Southwest University Chongqing 400715, China.

* Corresponding author, E-mail addresses: lancheng@swu.edu.cn
* Corresponding author, E-mail addresses: fydai@swu.edu.cn (F. Y. Dai)

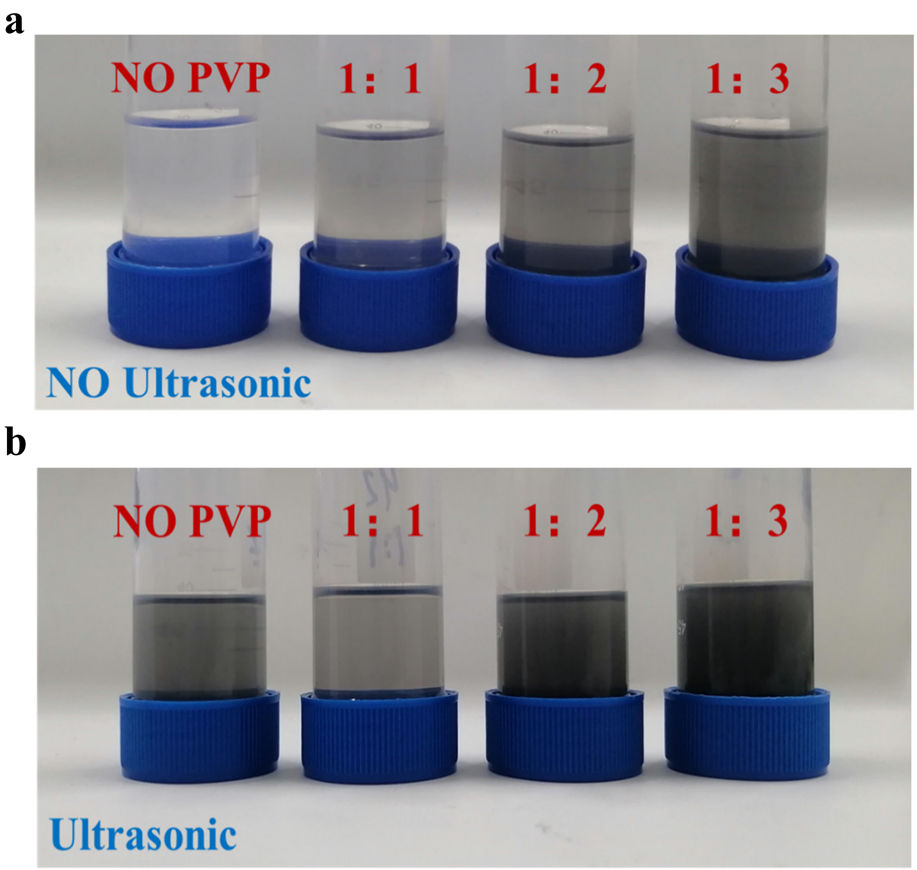

Figure S1 a) The graphene solution was stood for 2 h without ultrasound, b) The graphene solution was sonicated for 2 h and then stood for 24 h.

Table S1 The absorbance of graphene solution

	NO PVP	1： 1 (Gr： PVP)	1： 2 (Gr： PVP)	1： 3 (Gr： PVP)
NO Ultrasound	0.009	0.073	0.206	0.961
Ultrasound 2 h	0.520	0.255	0.803	1.116

Graphene was poorly dispersed in ethanol. PVP was selected as the dispersant for the graphene solution to obtain uniformly dispersed graphene solution.

In this study, a high concentration of graphene solution was required. The 10 mg/mL graphene solution was selected to the dispersity test. The graphene concentration was too high to observe the degree of dispersion. The samples were

stood for 24 h, diluted the supernatant 100-fold. Figure S1a shows the solution without ultrasound. And Figure S1b are the images of Graphene/PVP suspension with different ratio (sonicated for 2 h). Obviously, graphene solution with the ratio of 1:3 disperse best. It can be observed that with the increase of PVP ratio, the dispersion degree of graphene becomes better, but were worse than the samples which after ultrasound. The samples were further tested by UV spectrophotometry. The results (Table S1) showed that the solution with high PVP content dispersed better after ultrasound. Excessive PVP may cause the needle tube be blocked during electrostatic spinning and reduce the conductive performance of the sensor. Therefore, the plan with a ratio of 1:3 and sonicated for 2 h was selected to continue the subsequent experiment.

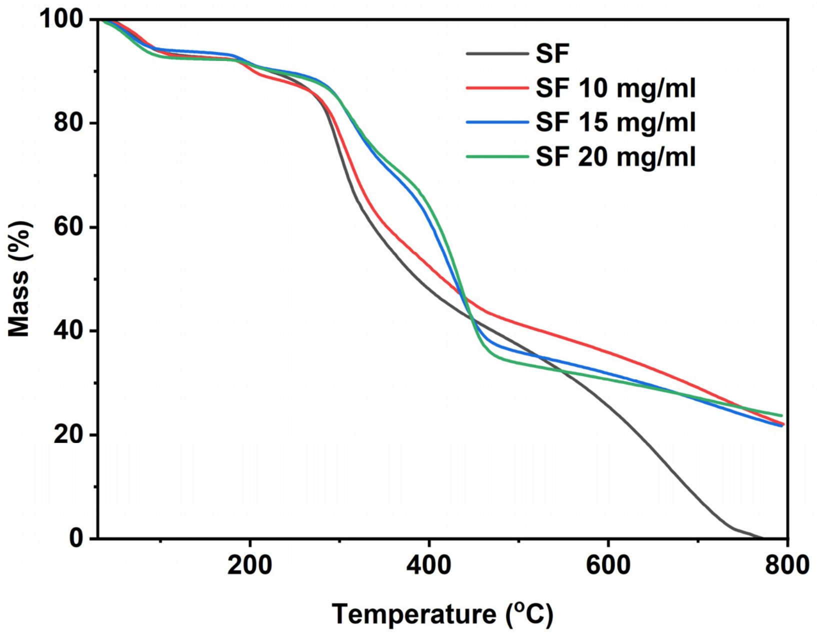

Figure S2 the TG curves of nanofiber membranes

It can be seen that the onset temperature of nanofiber membranes after introduce Gr is higher compared to that of SF nanofiber membrane. Furthermore, the mass

remaining of nanofiber membranes after introduce Gr is also larger than the SF nanofiber membrane. This result suggested that the SF/ Gr nanofiber membranes has good thermal stability.
